# Supplementary material for: Rethinking IL-1 Antagonism in Respiratory Viral Infections: A Role for IL-1 Signaling in the Development of Antiviral T Cell Immunity
Source: Int J Mol Sci. 2023 Oct 30;24(21):15770. doi: 10.3390/ijms242115770 (PMC10650641; doi:10.3390/ijms242115770)
Supplement: Supplementary file 1 [file ijms-24-15770-s001.zip › ijms-2655441-supplementary.pdf]

Supplementary Materials for

Rethinking IL-1 antagonism in respiratory viral infections:

A role for IL-1 signaling in the development of antiviral T cell immunity

Bram Van Den Eeckhout, Marlies Ballegeer, Jozefien De Clercq, Elianne Burg, Xavier Saelens, Linos Vandekerckhove and Sarah Gerlo*

* Correspondence: Prof. Dr. Sarah Gerlo
Corneel Heymanslaan 10, Medical Research Building (MRB) 2, Entrance 38, 9000 Ghent, Belgium
sarah.gerlo@ugent.be

These Supplementary Materials include:

- Supplementary Figures S1 – S6

A

BM-DC/OT-I CD8 T cell coculture experiments

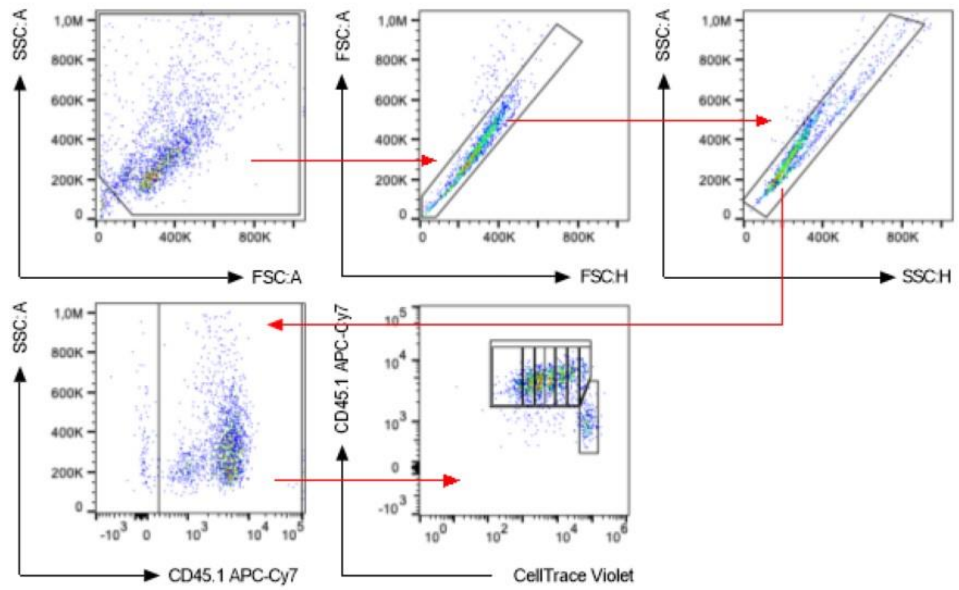

B

DC-OVA$_{257-264}$ experiments

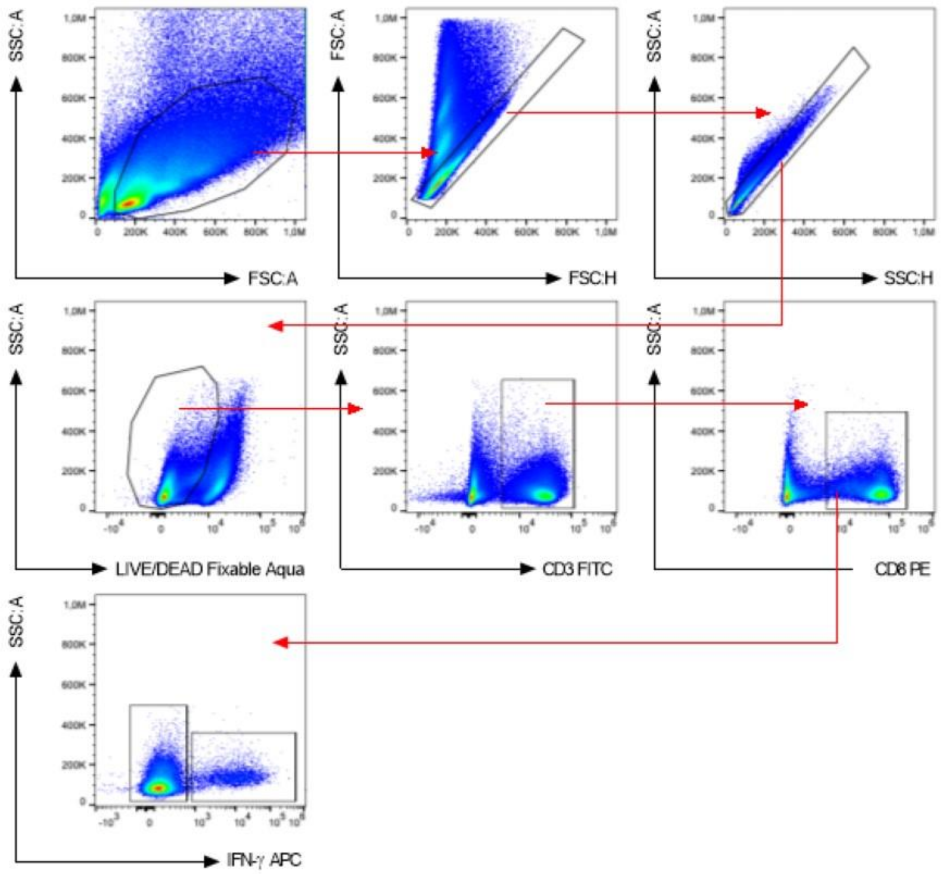

Supplementary Figure S1. (**a**) Gating strategy for the BM-DC/OT-I co-culture experiments. (**b**) Gating strategy for the *in vivo* DC-SIINFEKL immunization experiment.

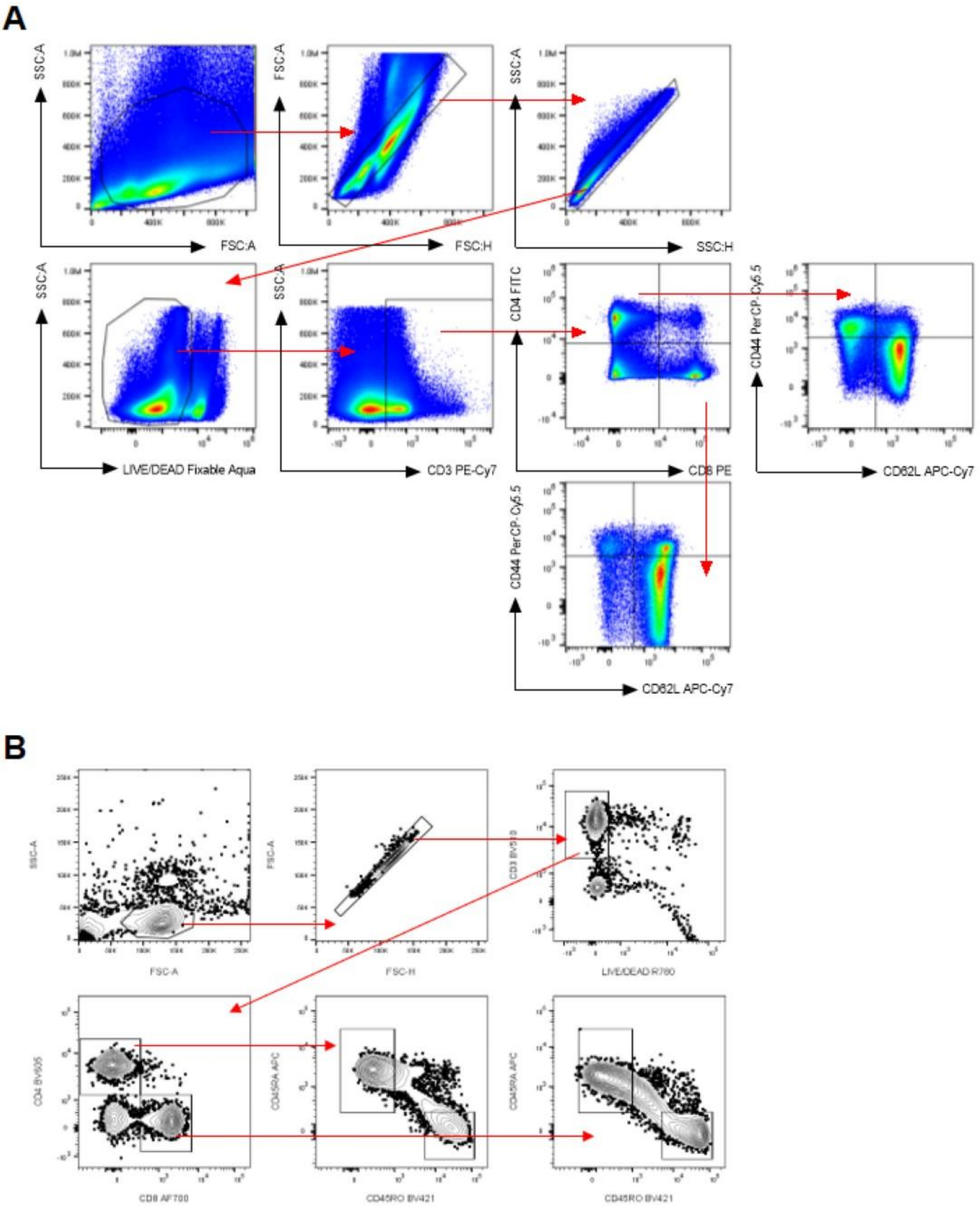

Supplementary Figure S2. (**a**) Gating strategy for the identification of CD4 and CD8 Tn cells and Tem cells for detection of IL-1R1 expression levels. (**b**) Gating strategy for the phosflow experiment on PBMCs.

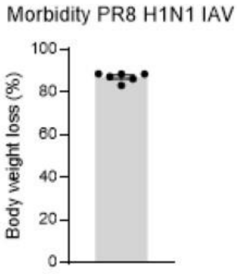

Supplementary Figure S3. Morbidity of PR8 IAV infection in C57BL/6 mice, assessed by body weight loss (%) 7 days post-inoculum.

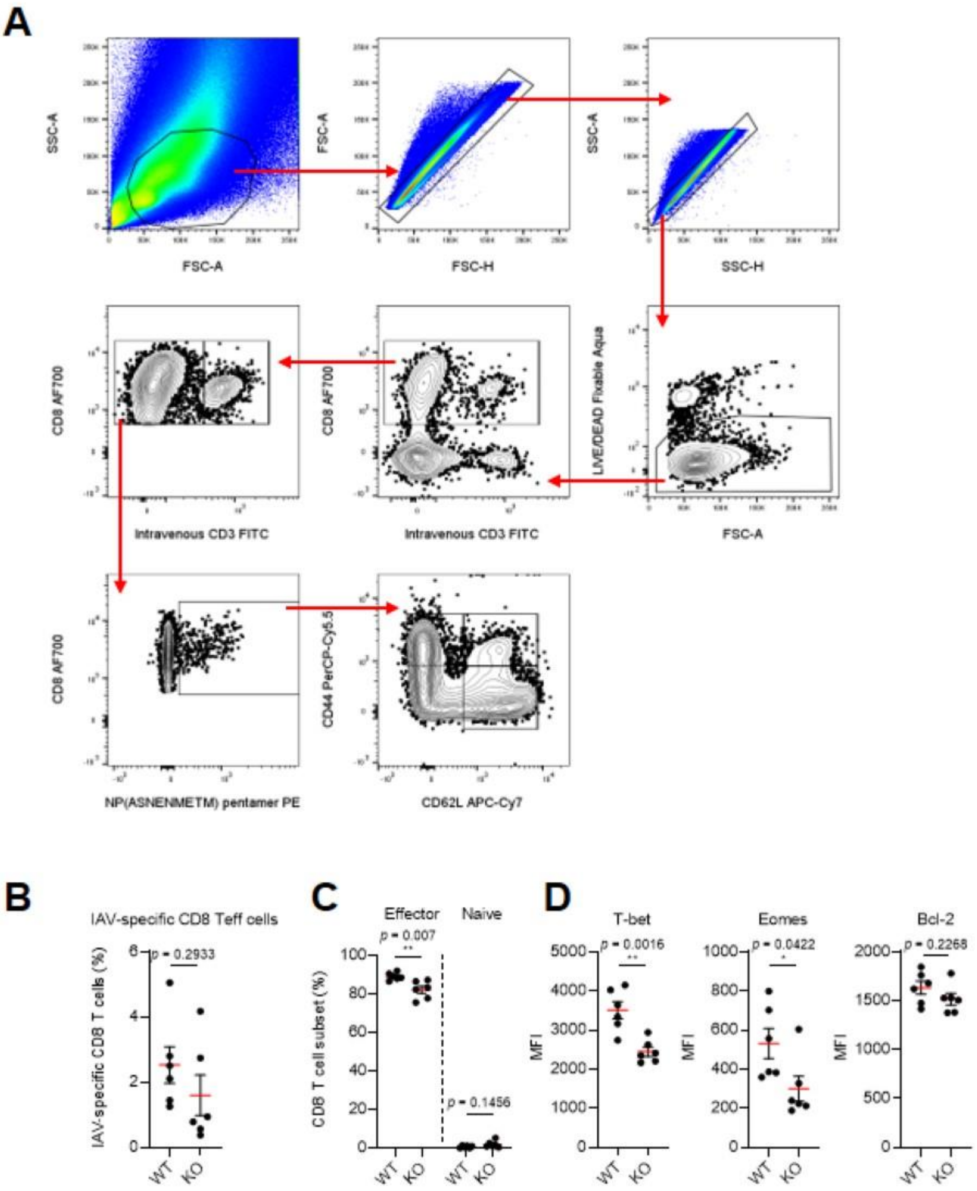

Supplementary Figure S4. (**a**) Gating strategy for the identification of antiviral CD8 Teff cells one week post-infection of WT and KO mice with PR8 IAV. (**b**) Frequencies of antiviral CD8 Teff cells within the total intravascular CD8 T cell population in lungs of WT and KO mice one week after PR8 IAV infection. (**c**) Frequencies of antiviral CD8 Teff cells that adopt an effector and a naive phenotype based on surface expression of CD44 and CD62L. (**d**) Expression of T-bet *(left)*, Eomes *(middle)* and Bcl-2 *(right)* in antiviral CD8 Teff cells. The mean ± SEM of an experiment with $n = 6$ mice/group is shown. **, $p < 0.01$; *, $p < 0.05$; ns, $p \geq 0.05$ by unpaired Student's *t*-test.

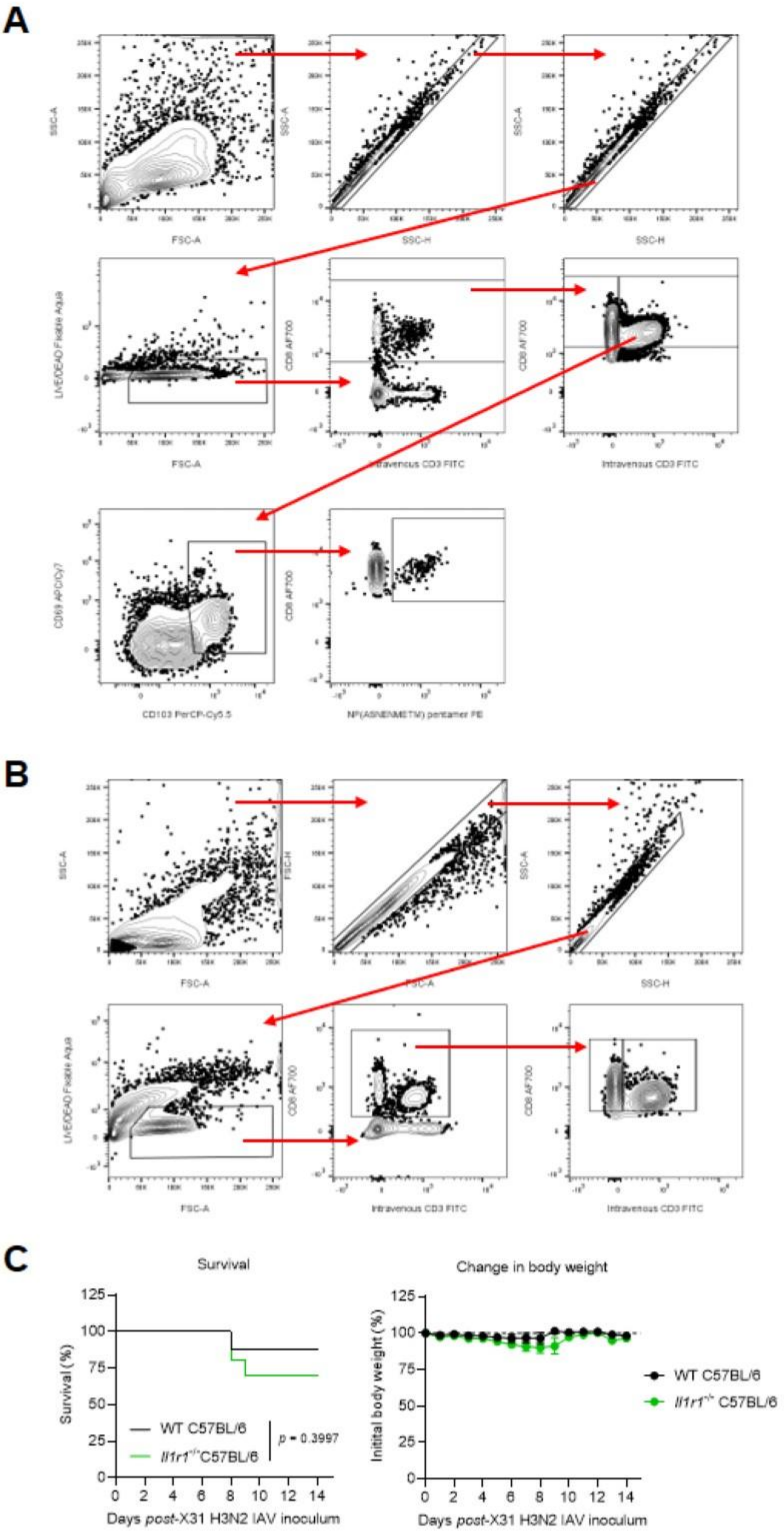

Supplementary Figure S5. (**a**) Gating strategy for the identification of antiviral CD8 Trm cells six weeks post-infection of WT and KO mice with PR8 IAV. (**b**) Gating strategy for the identification of polyfunctional CD8 Trm cells six weeks post-infection of WT and KO mice with PR8 IAV.

Representative image from lung single-cell suspensions stimulated for 6h *ex vivo* with PR8 IAV NP peptide pools. (**c**) Change in body weight over time of WT and KO mice challenged with a high inoculum (2xLD50) of X31 (H3N2) IAV. Mice were challenged six weeks after initial PR8 IAV infection and received a daily treatment with FTY720 (1 mg/kg) starting three days before X31 (H3N2) infection. Shown is a Kaplan–Meier curve of two independent experiments with n = 8 – 10 mice combined. ns, $p \geq 0.05$ by log-rank (Mantel-Cox) testing.

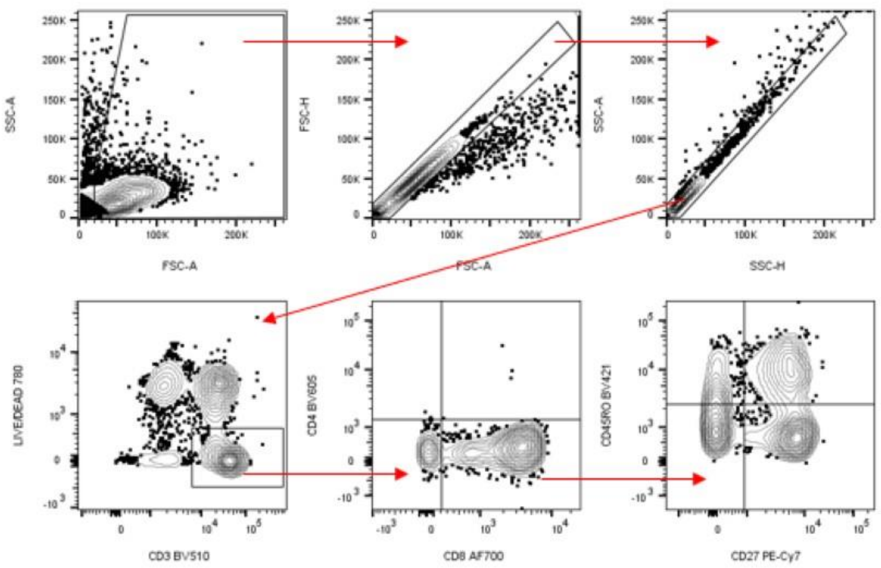

Supplementary Figure S6. Gating strategy for the human moDC/CD8 T cell co-culture experiment.
